# A Hospital Equerry for the King

**Published:** 1914-07-18

**Authors:** 


### The Hospital

The Workers' Newspaper of Administrative Medicine and Institutional Life, Administration, National Insurance and Health.

No. 1465, Vol. LVI. SATURDAY, JULY 18, 1914. PRICE ONE PENNY.

### A HOSPITAL EQUERRY FOR THE KING.

In one sense the experience and opportunities of King George and Queen Mary at Glasgow on Tuesday the 7th instant were unique. No other reigning monarch of any nation in the world has ever had the privilege of opening on the same day two such fine hospitals as the Royal Infirmary and the Royal Hospital for Sick Children, Glasgow, in fact are. The Royal Infirmary, mainly through an accident due to the obstinacy of the Scottish character, has been built upon a site which has overcome and removed the objections sometimes justly raised against great pavilion hospitals on administrative grounds. The Royal Hospital for Sick Children is so designed that anyone viewing it from the outside could never believe that it contains accommodation for 200 cots, much less that it presents practically the newest, latest, and most efficient structural and working equipments which knowledgeable and expert technical authorities can provide. Why, seeing that it has cost £700 per bed, and has so fine a site, an adequate sum was not used by the architect to present Glasgow with a group of buildings of architectural beauty we cannot imagine. Had the Royal Infirmary buildings been placed upon the site occupied by the Children's Hospital, the imposing attractiveness of the architectural features which Mr. Miller has worked into his design for them would have caused a multitude of people interested in architecture to go to Glasgow for the pleasure of studying the façades in Cathedral Square and in Castle Street.

But the hospital fare presented to the King and Queen of England on the 7th of this month goes further and deeper. Not only did their Majesties open two hospitals, but they visited the great hospital of the University, the Western Infirmary, and it would have been a genuine pleasure to His Majesty, we are convinced, had it been possible for the King to have seen some of the marked administrative efficiencies of this institution. To ensure that such efficiencies are not missed by His Majesty, it is essential that King George should have on his staff a hospital equerry whose duty it would be to keep himself in touch and up to date with every matter of special interest which hospital progress may anywhere present. Such an equerry would have to be a man of affairs, and be possessed of sound, impartial judgment, so that he could appraise an atmosphere and enter into and realise the joy of being associated with hospital work of the highest and best type. It should be the proud privilege of any such equerry to provide that not a moment of their Majesties' time, when visiting a hospital, should be wasted. With this end in view the King and Queen should have brought to their notice everything capable of providing food for thought from its demonstrable excellence.

We have ventured to call attention to this suggestion of an extra equerry to the King, because we greatly appreciate the genuine interest taken by His Majesty in our voluntary hospitals, and the surprising amount of information he has acquired about them and their management. King George is, in fact, a knowledgeable inspector of hospitals and kindred institutions. Indeed effective changes have been introduced into the management of more than one institution as the direct outcome of the King's visits. His father was heart and soul in the cause of the voluntary hospitals, and nothing was too much trouble or too difficult for him when the benefit of the sick was the end to be gained. We feel, therefore, that King George might set an example for good to the whole of the governing authorities throughout the world should he ever see his way to appoint a knowledgeable hospital equerry. The King is the titular head of many things in this nation. Having identified himself closely with our voluntary hospital system, it would be but natural for His Majesty to desire to provide himself with first-hand knowledge of everything calculated to secure the interest and effectiveness of the voluntary hospitals.

